# Disequilibrium reaction pathways and the twin-mediated growth of tabular forsterite during contact metamorphism of quartz-bearing dolomite

**DOI:** 10.1007/s00410-024-02096-2

**Published:** 2024-02-20

**Authors:** Marisa D. Acosta, Lukas P. Baumgartner

**Affiliations:** Institute of Earth Sciences, University of Lausanne, Géopolis, CH-1015 Lausanne, Vaud Switzerland

**Keywords:** Reaction overstepping, Contact aureole, TPRE, Twinning, Olivine, Disequilibrium

## Abstract

The forsterite zone of the Ubehebe Peak contact aureole, Death Valley, USA consists of an outer zone of tabular/jack-straw olivine and an inner zone of subequant polyhedral olivine. Subequant polyhedral forsterite crystals close to the intrusion are small and tabular forsterite crystals farther away are larger. To investigate the formation of the two morphologies, forsterite growth experiments were conducted in cold seal pressure vessels in the CaO-MgO-SiO_2_-CO_2_-H_2_O system. Forsterite precipitation follows a disequilibrium reaction pathway made of three reactions: [1] tabular forsterite growth from quartz and dolomite, [2] forsterite growth from tremolite dissolution, and [3] subequant polyhedral forsterite growth from tabular forsterite dissolution. Initially, quartz reacts with dolomite to simultaneously form twinned tabular forsterite and tremolite. As quartz reacts away, forsterite precipitation continues at a slower rate through tremolite dissolution. A second generation of forsterite then precipitates on top of some tabular forsterite but has different habit and tracht. Once all the tremolite reacts away, subequant polyhedral forsterite precipitation continues at an even slower rate through dissolution of tabular forsterite. The tabular morphology of jack-straw olivine is a consequence of twin-mediated unidirectional growth; the abundance of twins being due to rapid nucleation and growth at initially high reaction affinities. Twin junctions are preferential nucleation centers for steps, so faceted growth is enhanced on {100}. This phenomenon is the twin plane re-entrant effect. Subequant polyhedral forsterite in the Ubehebe Peak inner contact aureole recrystallized and ripened from tabular forsterite. In the outer contact aureole, conditions were not conducive to recrystallization and ripening so well-developed tabular forsterite persists.

## Introduction

Crystallization requires supersaturation and is intrinsically a manifestation of disequilibrium. The degree of supersaturation, which correlates positively with the magnitude of reaction affinity, is a measure of the driving force for a reaction. The kinetics and mechanics of nucleation and growth for a given phase are dictated by supersaturation (e.g., Sunagawa 2007; Dhanaraj et al. 2010). At high supersaturations, nucleation rates are relatively fast and growth rates are relatively slow; homogeneous nucleation is more likely to occur, twinning is generally more frequent, growth surfaces are rough, and growth happens by way of the continuous growth mechanism. At lower supersaturations, nucleation rates are relatively slow and growth rates are relatively fast; heterogeneous nucleation is more likely to occur, twinning is generally less frequent, growth surfaces are faceted, and growth happens by the two-dimensional nucleation and growth mechanism or by the spiral growth mechanism. At relatively high supersaturations, a large number of small crystals will form. At relatively low supersaturations, a small number of large crystals will form. Many rocks texturally record complex crystallization histories and evidence of disequilibrium, which complicates (or precludes) the application of equilibrium thermodynamics. However, because crystallization is a rate dependent process, rocks preserving evidence of reaction far from chemical equilibrium can be used to constrain the timescales of geologic processes.

Marbles formed by contact metamorphism of carbonate rocks are an excellent natural laboratory to study the kinetics of crystal growth because they have relatively simple metamorphic histories, lateral pressure effects can generally be assumed to be secondary to temperature effects, and they have a well-defined equilibrium phase petrology. In this contribution, we focus on forsterite formation during prograde contact metamorphism of quartz-bearing dolomites from the Ubehebe Peak contact aureole in Death Valley. We combine field and thin section observations, and geochemical and crystallographic data of Ubehebe forsterite with a series of simple experiments to illuminate overstepped reaction pathways and suggest that tabular “jackstraw” olivine is a consequence of twinning.

## Background

### The Ubehebe Peak contact aureole

The Ubehebe Peak contact aureole is at the southernmost edge of the Last Chance Mountains in Death Valley, California, close to the western extent of the Basin and Range physiographic region. In the late Paleozoic to early Mesozoic, compressional thrusting folded and tilted Paleozoic marine shelf carbonate strata. This early regional deformation set the stage for subsequent local deformation due to magma intrusion and emplacement in the Ubehebe Peak region. The 173 ± 1 Ma Ubehebe Peak quartz monzonite pluton is part of the composite Hunter Mountain batholith (Roselle et al. 1997). The country carbonates consist of, in order of oldest to youngest: the Ordovician Ely Springs dolomite, the Silurian Hidden Valley dolomite, and the Devonian Lost Burro formation. All of the country rocks are siliceous dolomites. The majority of outcropping contact metamorphism is hosted in the Lost Burro formation, which consists of light- and dark-gray dolomite and limestone with sandstone layers at the top and a distinctive basal quartzite (McAllister 1956).

**Fig. 1:**
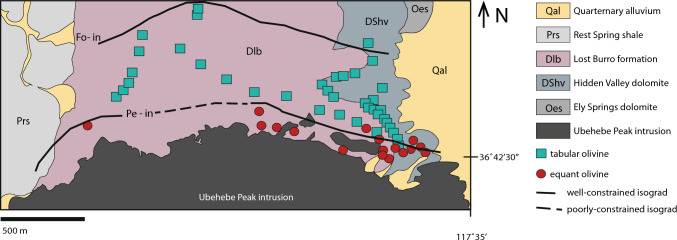
The forsterite (Fo) zone of the 173 ± 1 Ma Ubehebe Peak contact aureole can be subdivided into two parts: an inner forsterite (Fo) zone where olivine occurs as small (< 1 mm) subhedral subequant polyhedral crystals (red dots) and an outer forsterite zone where olivine occurs as larger (5–20 mm long) euhedral tabular crystals (teal squares). The difference in morphology broadly coincides with the periclase (Pe) -in isograd. Modified from Roselle et al. (1997)

The prograde contact aureole consists of an outermost tremolite zone, a middle forsterite zone, and an inner periclase zone. Pressure was probably between 1.5 and 2 kbar (Roselle et al. 1997). The focus of this contribution is forsterite habit in the contact aureole, which is subequant polyhedral near the igneous contact, but tabular in the outer edge of the forsterite zone (Fig. 1). As detailed below, this morphological change is observed in other contact aureoles and is accompanied by a change in crystal size distribution.

**Fig. 2:**
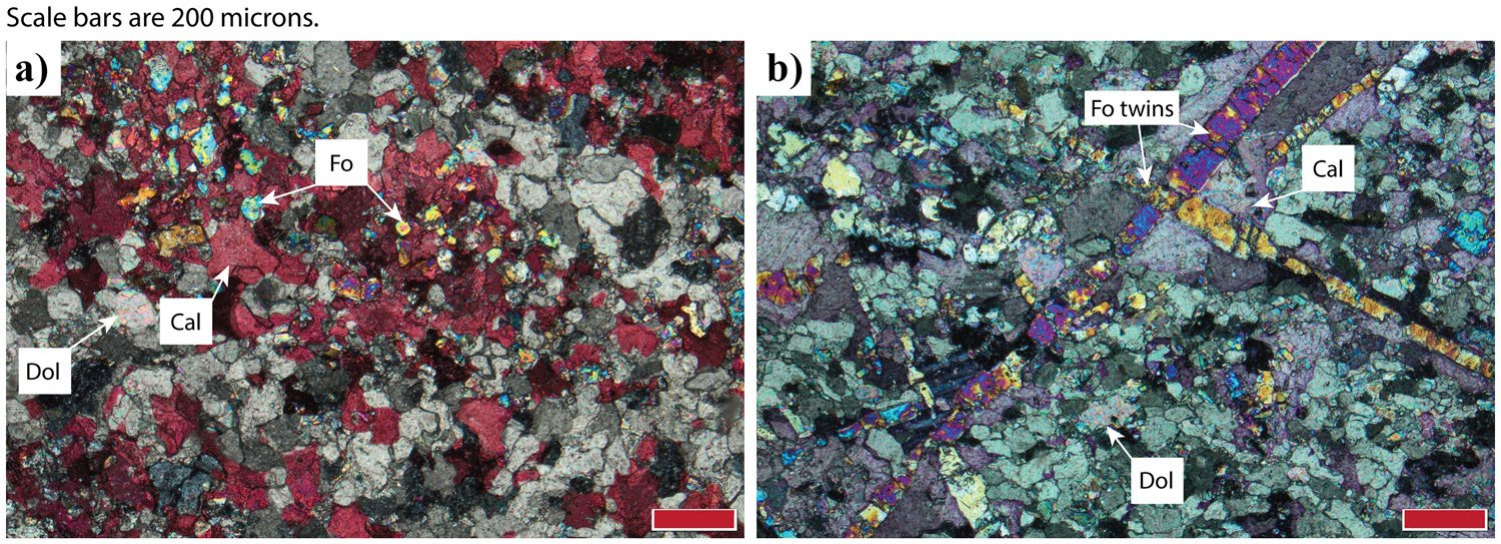
Typical thin section textures for subequant polyhedral and tabular forsterite as seen under cross polarized light. **a** Close to the intrusion, forsterite (Fo) is present as small, subequant polyhedral crystals (sample 1913/119). **b** Farther from the intrusion, forsterite is present as large, tabular crystals that are very obviously twinned (sample 1913/100). All forsterite is surrounded by a calcite (Cal) corona, but some tabular forsterite is directly in contact with unreacted dolomite (Dol)

At the deposit scale, olivine immediately adjacent to the pluton contact is subequant polyhedral and < 1 mm. There is a transition that is discrete on the scale of a hand sample, to forsterite that has a tabular morphology (*b* ⪆ *c* >  > *a*) and is larger than subequant polyhedral forsterite (> 1 mm in the middle forsterite zone and up to 20 mm at the outer forsterite zone). Forsterite precipitation is tightly linked to the initial silica content of the host rock because it preferentially occurs at the contact of the Hidden Valley Formation with the silica-rich Lost Burro Formation (Fig. 1). In thin section, forsterite of both habits is surrounded by coronas of calcite (stained pink with alizarin red S in Fig. 2) and twins are abundant. Fluid and calcite inclusions are plentiful in subequant polyhedral forsterite.

### Forsterite structure and twin laws

**Fig. 3:**
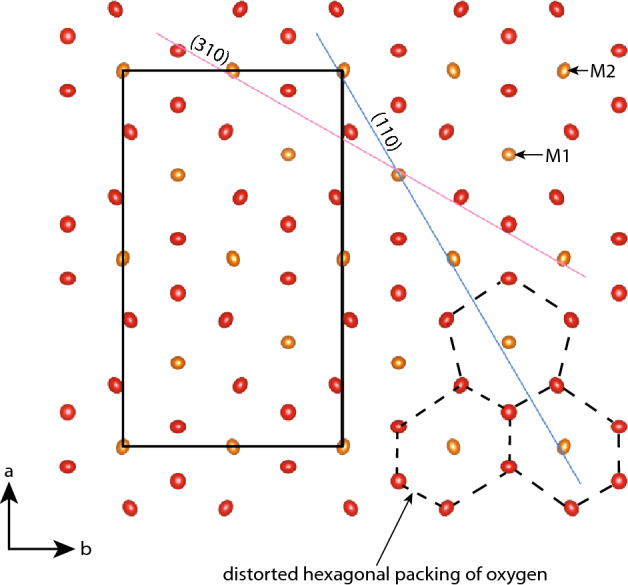
The structure of olivine viewed along < 001 > in the Pnma setting. The unit cell is indicated by the black rectangle. Two possible twin planes, (310) and (110), are also shown. The observed twins may be due to reflections across {310} or {110} or rotations around < 310 > or < 110 > . The twin angles are ~ 60° for either twin law. Pseudohexagonal {110} cyclical twins are common in many orthorhombic minerals such as aragonite or chrysoberyl. In the case of olivine, it is the distorted hexagonal packing of oxygen atoms (red displacement ellipsoids) that lends itself to pseudohexagonal symmetry. There are two cation sites (yellow displacement ellipsoids): the M1 and the M2. The M1 sites are centers of symmetry and the M2 sites are located on mirror planes. The M2 site is more prone to distortion than the M1 site, which is why the M1 displacement ellipsoids in the figure are more spherical than the M2 displacement ellipsoids

Olivine is a nesosilicate with the structural formula M1M2SiO_4_, where M1 and M2 refer to unique octahedrally coordinated cation sites (Fig. 3). Twinning in olivine is rarely reported (see review in Azevedo and Nespolo 2017), but is known to be more common in Ca-rich systems. There are three olivine twin laws, described here in the standard *Pnma* setting: {110}, {310}, and {120}. Twinning via reflection across {120} is a hybrid twin (Nespolo and Ferraris 2006) with a ~ 32° dihedral angle. At Ubehebe Peak, we observe that olivine is pseudohexagonally twinned according to either {110} or {310} twin laws, both of which have a dihedral angle of ~ 60°. The pseudohexagonal symmetry is due to the hexagonal packing of oxygen ions (Fig. 3). A fourth possible twin law is the {210} which would have a 90° dihedral angle (Welsch et al. 2013; also reported as {100} twinning in Dodd and Calef 1971), but this relationship is more likely to be an intergrowth instead of a true twin (Azevedo and Nespolo 2017).

Ubehebe Peak tabular olivine twins have a branching appearance that is reminiscent of dendritic growth (Fig. 2B), but the tabular individuals themselves do not appear to have dendrite characteristics. For example, the facets of individual twin members appear to have formed by 2D nucleation and growth on smooth faces; there is not evidence of interfacial instabilities on the tabular crystals.

### Forsterite in contact aureoles

Contact metamorphism of siliceous dolomites generally produces the following sequence of minerals, in order of decreasing grade/temperature: periclase, forsterite, diopside, tremolite, and talc (Moore and Kerrick 1976). The sequence can be incomplete in any given contact aureole. The forsterite zone of dolomitic contact aureoles can be zoned with respect to olivine habit and crystal size distribution. Tabular olivine is found in many dolomitic contact aureoles: the Ubehebe Peak marble (Roselle et al. 1997); the Alta stock aureole in Utah, USA (Beno et al. 2020); the Beinn an Dubhaich contact aureole, Isle of Skye, Scotland (Ferry et al. 2011); and the Twin Lakes pendant, California, USA (Ferry et al. 2011). Similar olivine shapes are also common in deserpentinized peridotite contact aureoles: the Bergell contact aureole, northern Italy (Clément et al. 2019; Lafay et al. 2019); the Bear Mountain pluton in the Klamath mountains, California (Snoke and Calk 1978). Tabular forsterite also occurs in regionally metamorphosed peridotites, for example, and the ultramafic Caledonian nappes in Norway (Bakke et al. 1986). The habit and tracht of tabular forsterite in ultramafic rocks are not the same as those found in dolomitic rocks (Dilissen et al. 2021) but the relative ratio of elongation along the crystallographic axes are the same (*Pnma*: b ⪆ c >  > a).

At Ubehebe Peak, Roselle et al. (1997) documented linear logarithmic relationship between the number of forsterite crystals normalized to the moles of forsterite per unit volume correlated with the distance from the contact; crystal number density is inversely correlated to crystal size. The number of forsterite crystals per mole of forsterite is a proxy for the integral of the nucleation rate over the reaction duration and the moles of forsterite per unit volume is a proxy for the integral of the growth rate over the reaction duration. Thus, the ratio of the number of forsterite crystals to the number of moles of forsterite is proportional to the ratio of the time-integrated nucleation and growth rate. Roselle et al. (1997) proposed that this relationship was caused by variation in reaction affinity across the contact aureole. Reaction affinity is a measure of the magnitude of driving force in a system; it is conceptually similar to supersaturation or log(*Q/K*). Rapid heating and infiltration of volatile-rich fluids derived from magma into dry country rock close to the pluton resulted in large reaction overstepping and nucleation was fast relative to growth in the region of the forsterite zone dominated by numerous small crystals of forsterite. Farther away the reaction overstepping was less dramatic due to cooler, more equilibrated fluids and so growth was faster than nucleation in the outer region of the forsterite zone dominated by fewer, but larger forsterite crystals. Reaction affinity also depends on the *a*_*CO*2_ and *a*_*SiO*2_ of the fluid.

Ferry et al. (2011) studied vein forsterite and also concluded that the size, shape, and modal abundance of olivine was a function of reaction affinity. However, the systems from the Ferry et al. (2011) study were “fluid-buffered”, so the *a*_*CO*2_ and *a*_*SiO*2_ of the infiltrating fluid dictated the spatio-temporal variation in reaction affinity. The Ubehebe Peak system was “rock-buffered”, so the thermal gradient of the contact aureole exerted the strongest control on the spatio-temporal variation in reaction affinity.

In basaltic systems, it is well-established that olivine morphology is a function of cooling rate and the degree of under cooling (Donaldson 1976; Faure et al. 2003, 2007). There are systematic changes in olivine habit, listed in order of increasing supersaturation, from: polyhedral subequant polyhedral crystals, polyhedral tabular crystals, skeletal hopper and swallowtail crystals, and dendritic crystals at the highest reaction affinities. The occurrence of olivine with skeletal or hopper growth features is rare in Ubehebe; features that were interpreted as skeletal growth were found by Beno et al. (2020) in forsterite dissolved out of marble from the Alta stock.

It has been suggested that the habit change from subequant polyhedral to tabular could be because the subequant polyhedral crystals grew above the roughening temperature of forsterite and that the tabular crystals grew below it (Roselle et al. 1997). An alternative hypothesis that has been put forward is that crystals precipitated at high reaction affinities closer to the intrusion will be smaller than those produced at low reaction affinities farther from the intrusion and so able to texturally equilibrate more readily via surface and volume diffusion where atoms dissolve from some faces and then diffuse to and reprecipitate on others (Holness 2000; Ferry et al. 2011).

Müller et al. (2009) concluded that tabular forsterite formed via the metastable reaction:

$$SiO_{2\left( {qz} \right)} + 2MgCa\left( {CO_3 } \right)_{2\left( {dol} \right)} \to Mg_2 SiO_{4\left( {fo} \right)} + 2CaCO_{3\left( {cal} \right)} + 2CO_2 \qquad \left[ {{\text{Reaction }}1} \right]$$

because they found that the oxygen isotopes of forsterite and calcite were in equilibrium with each other, but those of dolomite were not, which they concluded requires that throughout the reaction pathway dolomite was only a reactant and never a product.

## Methods

### Petrography

Petrographic observations were made on covered thin sections, in which the calcite had been stained with alizarin red S, on a standard petrographic microscope using both plane polarized light and under crossed polars. All of the samples examined were from the collection originally acquired and described by Roselle (1997).

### Electron probe microanalysis (EPMA)

EPMA analyses were acquired at the University of Lausanne on a Jeol JXA-8350F HyperProbe Schottky field emission gun microprobe. On peak count times were 30 s and background count times were 15 s for all elements except for Ba, Sr, and Co, which had 60 s on peak and 30 s off. Si *Kα* and Mg *Kα* were measured on a TAP crystal. Na *Kα*, Al *Kα*, and Sr *Lα* were measured on a TAPL crystal. Mn *K*_*α*_, Co *Kα*, Ba *Lα*, Ni *Lα*, and Fe *Kα* were measured on an LIFL crystal. Ti *Kα*, Ca *Kα*, and P *Kα* were measured on a PETL crystal. The Ca results were affected by secondary fluorescence due to coronal calcite and so were discarded; in general measuring Ca in olivine by EPMA is very susceptible to secondary fluorescence effects, even when the matrix is a basaltic glass (Gavrilenko et al. 2023).

### Laser ablation inductively coupled mass spectrometry (LAICPMS)

The data were acquired at the University of Lausanne on a RESOlution SE 193 nm ablation system equipped with a two volume S155 ablation cell (Australian Scientific Instruments) and interfaced to a NexION 5000 quadrupole ICP-MS (Perkin Elmer) operated in the single-quadrupole regime and optimised for high sensitivity. Ablation was carried out at a fluence of 8 J*/cm*^2^, a repetition rate of 10–12 Hz, and a beam size of 38–100 microns. The primary standard was the SRM 612 soda-lime-silica glass from NIST; elemental abundances in it were taken from the GeoREM database. Silicon was used as an internal standard. Raw data were reduced in LamTrace (Jackson and Sylvester 2008); the critical levels of detection were computed at a nominal level of 5% false detections using the square root transform test, as recommended by Ulianov et al. (2016).

### Raman spectrometry

Spectra were acquired at the University of Lausanne on a Renishaw inVia confocal Raman microscope with a 532 nm laser at 2.5 mW and a 3000 mm^−1^ grating. Baseline-corrected spectra consisted of 2 accumulations of 15 s exposures over 100–1300 cm^*−*1^.

### Single crystal X-ray diffraction

The X-ray crystal structure determination service unit of the Department of Chemistry, Biochemistry and Pharmaceutical Sciences of the University of Bern is acknowledged for measuring, solving, refining and summarizing the structures of subequant polyhedral and tabular forsterite presented herein.

A forsterite crystal immersed in parabar oil was mounted and placed into a stream of nitrogen at 173 K. Measurements were done on a Rigaku XtaLAB Synergy R, HyPix-Arc 100 area-detector diffractometer using mirror optics with mirror optics monochromated Mo *Kα* radiation (*λ* = 0*.*710773 Å). Unit cell constants and orientation matrices were calculated from least-squares refinement of reflection setting angles from 3*.*882^*◦*^ < *θ* < 44*.*932^*◦*^. A total of 3964 frames were collected over *ω* scans with 1 s exposure time (3 s for high angle reflections), a 0*.*5^*◦*^ rotation angle, and a crystal-detector distance of 47 mm.

CrysAlisPro was used for data reduction. Intensities were corrected for Lorentz and polarization effects, and a Gaussian based numerical absorption correction over a multifaceted crystal model with additional empirical absorption correction using spherical harmonics using SCALE3 ABSPACK in CrysAlisPro was applied.

Structures were solved via intrinsic phasing using SHELXT (Sheldrick 2015a, b) in OLEX2 (Dolomanov et al. 2009), which revealed the positions of all nonhydrogen atoms. Nonhydrogen atom positions were anisotropically refined. Refinement of the structure was carried out on *F*^*2*^ using full-matrix least-squares procedures, which minimized the function Σ*w*(*F*_*o*_^2^ *– F*_*c*_^2^). The weighting scheme depended on counting statistics and downweighted intense reflections.

All results are given in standard *Pnma* setting of the space group, rather than the historically conventional *Pbnm*; an important consequence of this choice is that the twin laws {011}_Pbnm_ and {031}_Pbnm_ are respectively re-indexed as {110}_Pnma_ and {310}_Pnma_. This choice emphasizes that pseudohexagonal twinning is common in minerals with this symmetry and not unique to olivine; {110} cyclical twinning is especially common in orthorhombic minerals.

### Scanning electron microscopy (SEM)

Energy dispersive X-ray spectra (EDS), secondary electron (SE) images, and backscattered electron (BSE) images were acquired at the University of Lausanne on a Tescan Mira II LMU Shottky field emission scanning electron microscope at variable operating conditions. For unpolished samples, BSE images were almost always acquired instead of SE images due to extensive charging of the angular, loosely compacted material.

### Experimental methods

**Fig. 4:**
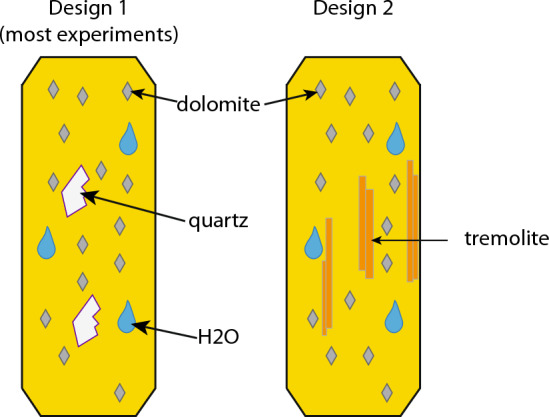
The two experimental designs: all but one experiment were Design 1, which was an Au capsule containing crushed natural dolomite powder, fragments of natural quartz, and 30 µL of water. A single experiment, forMor10, used tremolite as a starting material instead of quartz (Design 2)

Experiments were performed in vertically mounted cold seal systems. Two experimental designs were used (Fig. 4). The first, which was used in all experiments except forMor10, consisted of crushed and sieved natural Inyo Mts dolomite and quartz powders with 30 *µ*L H_2_O in thin-walled (0.1 mm) Au capsule. Experiments were heated to 650 °C by placing a pressurized Rene 41 bomb (1.7 kbar) into a pre-heated oven. N-type thermocouples from OMEGAT monitored temperature during runs in boreholes close to the capsule, with an error of about 3–5 °C. Experiments were cooled by removing the bomb from the oven and allowing it to cool conductively to 350 °C in approximately 5 min before being rapidly cooled to 75 °C with an air gun in 10 min. During heating and cooling, a pressure of 1*.*7 ± 0*.*15 kbar was maintained. After capsules were removed from the bombs, they were weighed to ensure no mass had been gained or lost during the run. The amount of *CO*_2_ that formed during the run was measured by gas loss after puncturing the capsule (Skippen and Hutcheon 1974, and references therein). A time series was done by varying the time at peak temperature of runs.

The maximum amount of CO_2_ produced during a given run was calculated before capsule assembly such that the final X_*CO*2_ of the fluid was less than 0.1 by making sure that the ratio of quartz to water in each capsule was about 0.005 g quartz to 0.03 g water. The amount of water added to each run was dictated by the volume of the capsules because it expands when heated, and the amount of quartz in each run was used to calculate CO_2_ production using the stoichiometry of Reaction (1).

## Results

### Petrography

Across the forsterite zone, forsterite in the siliceous dolomite is randomly oriented, surrounded by coronas of calcite, and serpentinite to varying degrees. Dolomite is granoblastic and ranges in size from approximately 20–150 µm. Where the dolomite is in contact with coronal calcite, it tends to have a slightly larger grain size. Coronal calcite is granoblastic, abundantly twinned, and ranges in size from approximately 75–300 µm (Fig. 2). Sparse, finer-grained (< 20 µm) calcite occurs interspersed throughout matrix dolomite. Subequant polyhedral forsterite is generally on the order of 50–150 µm in diameter (Fig. 2A), although isolated regions (1–5 mm) can have slightly larger grains (0.2–1 mm). These isolated regions with slightly larger grains are not proximal to veins and likely reflect a higher initial modal abundance of quartz in the dolomite protolith. Tabular forsterite ranges from about 2–20 mm in length and elongate parallel to the *a* axis. Olivine twins are readily observed under crossed polarized light. Tabular forsterite exhibits cellular and parallel growth textures sporadically throughout the contact aureole; cellular or parallel subequant polyhedral olivine is extremely rare. Olivine is serpentinized to varying degrees: some samples are wholly serpentinized, others are pristine, and most are somewhere in-between. Calcite coronas surrounding forsterite are irregular, and there are euhedral protrusions of forsterite directly in contact with dolomite (Fig. 2B). Subequant polyhedral forsterite contains abundant inclusions of calcite; some tabular forsterite also contains inclusions of calcite, but less abundantly.

### Electron probe microanalysis (EPMA)

**Fig. 5:**
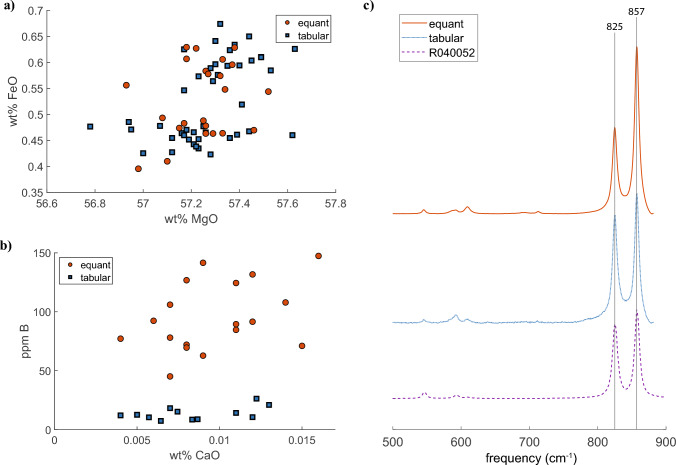
**a** Microprobe analyses show that there is complete overlap between subequant polyhedral and tabular forsterite with respect to the fayalite component; compositions across the contact aureole range from Fo99 to Fo98 with no spatial trends. **b** LAICPMS data shows that Ca concentrations in subequant polyhedral and tabular olivine completely overlap with one another, ranging from 0.004 wt% CaO to 0.02 wt% CaO. Subequant polyhedral olivine is systematically enriched in boron relative to tabular olivine; there is about an order of magnitude more B in subequant polyhedral olivine than there is in tabular olivine. **c** The characteristic doublets around 825 and 857 cm* − *1 shift downward systematically with Fe and Ca substitution into olivine (Kuebler et al. 2006; Mohanan et al. 1993). The purity of both subequant polyhedral and tabular forsterite, which is also shown in the microprobe and LAICPMS data, is indicated by the almost exact overlap of the doublet peaks. For reference, the Raman spectra of a synthetic forsterite is also shown (R040052; the RRUFF database)

For the elements measured, there were no systematic differences between tabular and subequant polyhedral forsterite populations. Compositions range from *Fo*_98*.*8_ to *Fo*_99*.*1_, where *Fo*# = *MgO/*[*MgO* + *FeO*]. There is complete overlap between the Fe and Mg concentrations of subequant polyhedral and tabular forsterite (Fig. 5A).

### LAICPMS

Subequant polyhedral forsterite is relatively enriched in B by an order of magnitude with respect to tabular forsterite (Fig. 5B). Ti, and Ni are also systematically enriched in subequant polyhedral forsterite, although to a lesser extent. Tabular forsterite is relatively enriched in Cr. There is complete overlap in measured concentrations of Ca, P, Co, Zn, Sr, Y, Zr, Mo, Ba, W, Pb, and Yb for subequant polyhedral and tabular forsterite.

### Raman spectrometry

The frequencies of the doublet made up of strong Si–O symmetric stretching bands around 825 and 855 cm* − *1 systematically shifts downwards with Ca and Fe substitution into forsterite (Mohanan et al. 1993; Kuebler et al. 2006). Average spectra of tabular and subequant polyhedral olivine (*n* = 10 each) have doublet peaks that are on top of each other (Fig. 5C). We interpret this to confirm that there is no significant structural modification of the lattices due to Ca or Fe incorporation; the forsterite is remarkably pure.

### Single crystal X-ray diffraction

There are small but significant and systematic differences between the structures of subequant polyhedral and tabular forsterite (see Tables 1 and 2). The unit cell of tabular forsterite is slightly larger (0.26 Å) than that of subequant polyhedral forsterite, has more variable bond angles, and a slightly higher distortion index. Bond angle variance and distortion index both describe warping of polyhedral. The distortion index describes the average deviation of cation-oxygen bond lengths from the average (Baur 1974; Zhang et al. 2019). Because the Raman and microprobe data suggest there is no lattice distortion due to impurity incorporation, this reflects structural disorder (i.e., bond length/angle disorder and defect density).

**Table 1:** Measured unit cell and polyhedral parameters from XRD analysis. Numbers in parentheses indicate the standard uncertainty of the last significant digit

*Pnma* unit cell	Subequant polyhedral	Tabular
a	10.18213(12) Å	10.18342(8) Å
b	5.97273(8) Å	5.97467(5) Å
c	4.74925(6) Å	4.75149(4) Å
Volume	288.827(6) Å^3^	289.093(4) Å^3^
**Silica tetrahedra**	**Subequant polyhedral**	**Tabular**
Volume	2.2077 Å^3^	2.2102 Å^3^
Average bond length	1.6354 Å	1.6361 Å
Bond angle variance (degree2)	49.9768	50.1105
Distortion index (bond length)	0.00660	0.00664
**M1 octahedra**	**Subequant polyhedral**	**Tabular**
Volume	12.3641 Å^3^	12.3754 Å^3^
Average bond length	2.1267 Å	2.1273 Å
Bond angle variance (degree2)	88.9699	88.7457
Distortion index (bond length)	0.03252	0.03251
**M2 octahedra**	**Subequant polyhedral**	**Tabular**
Volume	11.7346 Å^3^	11.7467 Å^3^
Average bond length	2.0918 Å	2.0925 Å
Bond angle variance (degree2)	94.6783	94.5945
Distortion index (bond length)	0.01158	0.01151

**Table 2:** Comparison of subequant polyhedral and tabular forsterite measured bond lengths from XRD analysis

Bond	Subequant polyhedral	Tabular
Si1-O1	1.6547(4) Å	1.6552(4) Å
Si1-O2	1.6366(3) Å	1.6143(4) Å
Si1-O3	1.6139(4) Å	1.6374(3) Å
Mg1-O1	2.0451(4) Å	2.0456(4) Å
Mg1-O2	2.0637(3) Å	2.1726(4) Å
Mg1-O3	2.1722(4) Å	2.2083(3) Å
Mg2-O1	2.0663(3) Å	2.0669(3) Å
Mg2-O2	2.1282(3) Å	2.0820(3) Å
Mg2-O3	2.0811(3) Å	2.1287(3) Å

### Scanning electron microscopy

Imaging of experimental products shows that tabular forsterite is faceted and has no discernible features of skeletal growth. Forsterite, tremolite, calcite, and dolomite were identified by EDS; no quartz was observed in any experimental run products.

### Experimental results

Table 3 contains an overview of experiments discussed herein.

**Table 3:** Most experiments were performed at a peak temperature of 650 *℃* and a pressure of 1.7 kbar using experimental design 1

Experiment	Time at 650℃	Result	Comment
forMor1	21 h	Tabular forsterite	Doped with Sr and Ba
forMor3	4 h	Tabular forsterite & tremolite	
forMor4	93 h	Tabular & subequant polyhedral forsterite	Forsterite structure confirmed with Raman
forMor5	71 h	Tabular & subequant polyhedral forsterite	
forMor6	503 h	Subequant polyhedral forsterite & partially dissolved tabular forsterite	
forMor7	< 1 min	Large tremolite crystals with trace tabular forsterite	Zero-dwell experiment
forMor8	–	Subequant polyhedral forsterite only	10 h at 800 ℃
forMor9	24 h	Starburst subequant polyhedral forsterite & partially dissolved tabular forsterite & tremolite	
forMor10	22 h	Stubby prisms of euhedral forsterite nucleated on tremolite surface	Capsule Design 2

The exceptions are forMor10 which used tremolite instead of quartz as a starting material and forMor8 which was held at 800 *℃*. All experiments had similar heating and cooling paths (see text of Experimental Methods section for details). After each run, capsule contents were imaged with an SEM which allowed for a comparison of mineral morphology and twinning type/frequency

**Fig. 6:**
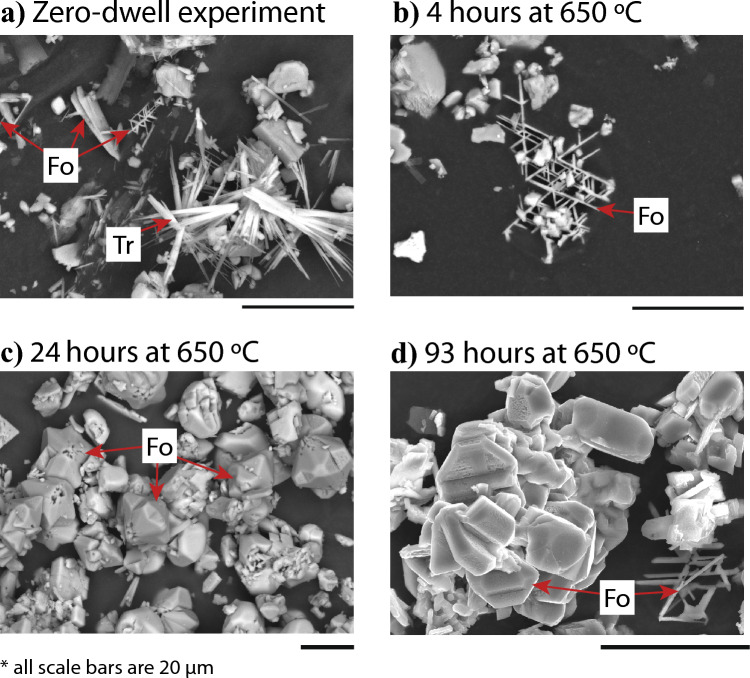
Scanning electron microscope (SEM) images of reaction products. **a** The first reaction products to form are acicular tremolite and small crystals of pseudohexagonally twinned tabular forsterite. **b** After only a few hours at 650 °C, almost all of the tremolite disappears and larger crystals of tabular forsterite form. **c** After a day at 650 °C, most of the forsterite has developed into a starburst shape, with voids at the centers of the crystal clusters. **d** After a prolonged dwell at 650 °C, the central voids are no longer visible and the crystals have developed flat faces. Trillings are observed throughout all experiments regardless of duration, while the habit and tracht evolve

**Fig. 7:**
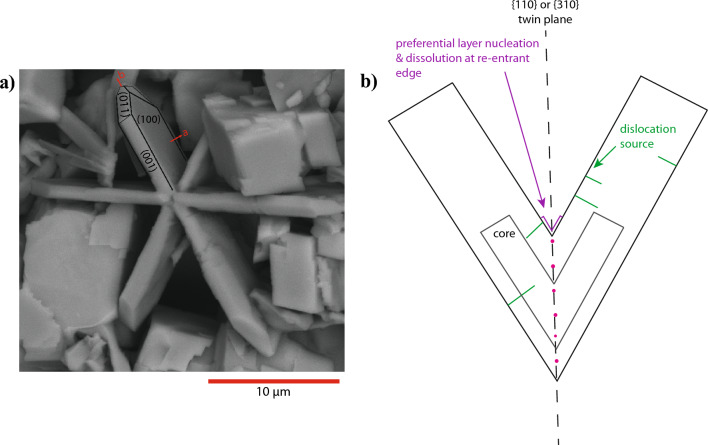
Scanning electron microscope image of twinned olivine with a schematic diagram. **a** The most well-developed face of the tabular olivine habit is the (100), which is annotated on the SEM-SE image on the left along with the prominent (001) and (110) faces. Crystals are elongate parallel to < 001 > and < 010 > and shortened in the < 100 > direction. **b** The TPRE describes the preferential nucleation and growth of layers at the re-entrant edges of twin boundaries. As the crystal grows in size, nucleation also occurs at dislocation sources which are always perpendicular to the established growth face and so unidirectional growth occurs. Although there are no significant impurities in the bulk of the twinned crystals, it is possible that impurities are enriched in the twin plane itself which would explain the correlation between Ca in the system and olivine twinning frequency

A zero-dwell experiment, wherein the capsule was isobarically heated to 650 °C and then immediately isobarically cooled, yielded a lot of acicular tremolite and a lesser amount of smaller tabular forsterite crystals (Fig. 6A). An otherwise identical experiment that was held at 650 °C for 3 h contained less tremolite but larger crystals of tabular forsterite (Fig. 6B). An experiment held at 650 °C for 24 h contained no tremolite and the many of the forsterite crystals had ripened into subhedral subequant polyhedral crystals with voids at the centers of the twinned clusters (Fig. 6C). Those that had not ripened into subequant polyhedral crystals displayed dissolution features. An experiment that was held at 650 °C for 93 h displayed subequant polyhedral twinned euhedral crystals and partly dissolved tabular forsterite crystals (Fig. 6D). An experiment that was held at peak temperature for 30 days exhibited exclusively euhedral twinned subequant polyhedral forsterite and no tabular forsterite. Both reticulated aggregate twins and stellate trillings are common in tabular and subequant polyhedral forsterite (Fig. 7A). The tracht of tabular forsterite consists primarily of {100}, {001}, and {011} faces (Fig. 7B).

No tabular forsterite was observed in forMor10, a run that used tremolite instead of quartz as starting material (Design 2; Fig. 4). Instead, stubby 10–15 micron long prisms of euhedral forsterite were sparsely nucleated on tremolite surfaces. After 22 h, the majority of tremolite was unreacted.

## Discussion

### Reaction pathways

**Fig. 8:**
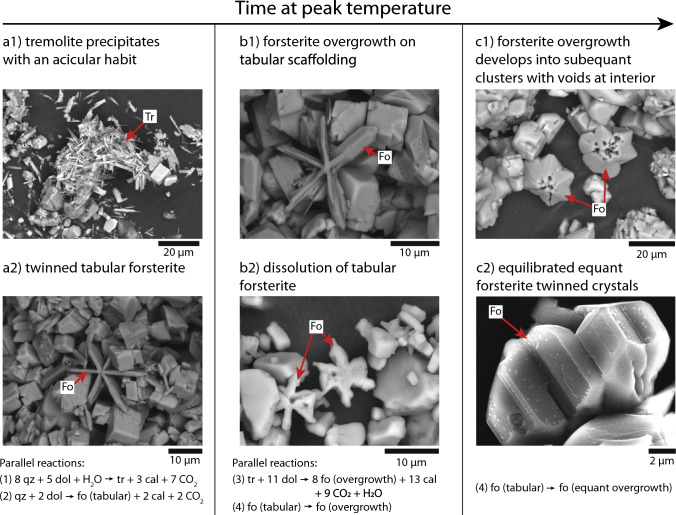
The variation of forsterite habit and tracht is due to a sequence of parallel reactions followed by a ripening reaction, shown here as a sequence of SEM-BSE images. Initially, quartz and dolomite react to form tremolite (**a1**) and twinned tabular forsterite (**a2**). Once quartz has been exhausted, tremolite reacts to form forsterite overgrowths on some tabular crystals (**b1**) while other tabular crystals dissolve (**b2**). Once all the tremolite has been exhausted, tabular forsterite dissolves to reprecipitate as subequant polyhedral forsterite. This results initially in twinned subequant polyhedral crystals with void-riddled interiors where the tabular scaffolding has dissolved out (**c1**) and ultimately in idiomorphic subequant polyhedral crystals with faceted surfaces (**c2**)

The results of the time series indicate that multiple reactions occur simultaneously in the capsules. Initially, tremolite precipitates (Fig. 8A1) by the reaction:

$$\displaystyle 8SiO_{2\left( {qz} \right)} + 5MgCa\left( {CO_3 } \right)_{2\left( {dol} \right)} \to Ca_2 Mg_5 Si_8 O_{22} \left( {OH} \right)_{2\left( {tr} \right)} + 3CaCO_{3\left( {cal} \right)} + 7CO_2 \qquad \left[ {{\text{Reaction }} { }2} \right]$$

At the same time, tabular forsterite (Fig. 8A2) forms via the reaction:

$$\displaystyle SiO_{2\left( {qz} \right)} + 2MgCa\left( {CO_3 } \right)_{2\left( {dol} \right)} \to Mg_2 SiO_{4\left( {tabular} \right)} + 2CaCO_{3\left( {cal} \right)} + 2CO_2 \qquad \left[ {{\text{Reaction }} { }3} \right]$$

which is the same reaction as Reaction 1, but we emphasize here that the olivine produced is tabular.

Perhaps in parallel with tremolite and tabular forsterite precipitation, tremolite dissolves to form forsterite:

$$\displaystyle Ca_2 Mg_5 Si_8 O_{22} \left( {OH} \right)_{2\left( {tr} \right)} + 11MgCa\left( {CO_3 } \right)_2 \left( {dol} \right) \to 8Mg_2 SiO_4 \left( {fo} \right) + 13CaCO_3 \left( {cal} \right) + 9CO_2 + H_2 O\; \left[ {{\text{Reaction }} { }4} \right]$$

The forsterite that precipitates from tremolite dissolution forms overgrowths on the pre-existing tabular forsterite (Fig. 8B1). Each arm of the tabular trillings are overgrown with forsterite that has a more normal olivine morphology in the sense that the overgrowths are lacking the tabular {100} faces. Even as some tabular crystals act as scaffolds for continued forsterite growth, other tabular crystals simply dissolve (Fig. 8B2) and act as another source of overgrowth forsterite:

$$\displaystyle Mg_2 SiO_{4\left( {tabular} \right)} \to Mg_2 SiO_{4\left( {equant} \right)} \qquad \left[ {{\text{Reaction }} { }5} \right]$$

After all of the tremolite in the capsule has been exhausted, trilled subequant polyhedral forsterite continues to morphologically ripen via Reaction (5). Initially, the centers of the trilled subequant polyhedral forsterite crystal clusters are riddled with voids due to the dissolution of their tabular interiors (Fig. 8C1). With time, these voids are filled in with either calcite or forsterite and the subequant polyhedral forsterite faces become flat (Fig. 8C2).

**Fig. 9:**
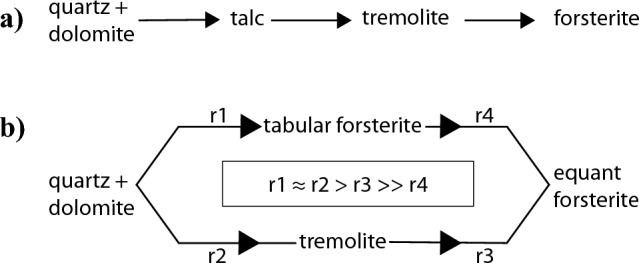
**a** The expected reaction pathway based on equilibrium thermodynamic phase petrology is that as the siliceous dolomite country rock is heated up by the nearby magma chamber, quartz and dolomite would react to form first talc, then tremolite, and lastly forsterite. However, the systematics of forsterite formation in these experiments suggests a more complex reaction pathway **b** where quartz and dolomite react to simultaneously produce tremolite and tabular forsterite which then dissolve at different rates to form subequant polyhedral forsterite. Note that all reactions include carbon dioxide and calcite as reaction products. The relative magnitude of the rate coefficients for each reaction rate law are also shown, where r1 is the rate constant for the precipitation of tabular forsterite, r2 is the rate constant for the precipitation of tremolite, and r3 and r4 are the rate constants for the precipitation of subequant polyhedral forsterite from the respective dissolution of tremolite and tabular forsterite

Reaction pathways are diagrammatically shown in Fig. 9. Initially quartz and dolomite are consumed to produce tabular forsterite and tremolite in parallel. After all the quartz has reacted out, tremolite and tabular forsterite dissolve to produce subequant polyhedral forsterite. After all the tremolite has reacted out, dissolution of tabular forsterite persists and forms subequant polyhedral forsterite with an equilibrium morphology. We note that all of the reactions discussed in this manuscript are actually occurring through ions in solution.

## Formation of tabular forsterite

### Pseudohexagonal twinning of tabular forsterite

**Fig. 10:**
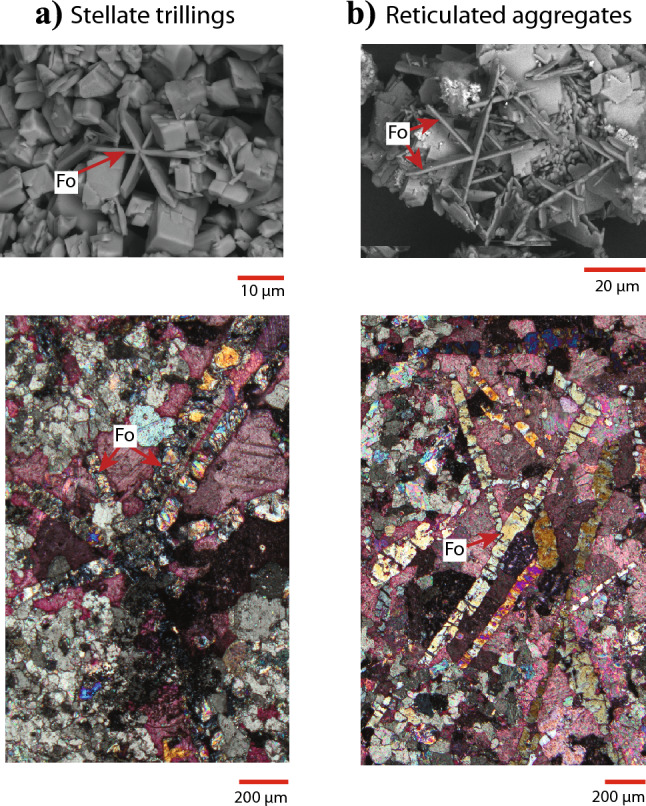
Two major types of twins occur in tabular forsterite. **a** Stellate trillings consist of cyclical sector twinning, where each twin member has developed in the same crystal faces and the same lengths. Stellate trillings are common in our experiments (SEM-BSE image; upper row) and scarce in Ubehebe (petrographic image; lower row). **b** Reticulated aggregates consist of a network of twins, each of which has developed the same crystal faces but different lengths. Both stellate trillings and reticulated aggregates could be due to either {310} or {110} twin laws. Reticulated aggregates are common in both our experiments (SEM-BSE image; upper row) and in Ubehebe samples (petrographic image; lower row. All petrographic images were taken under crossed polars

The most striking characteristic of tabular forsterite is the ubiquitous pseudohexagonal twinning. There are two apparently distinct types of twins: stellate trillings (Fig. 10A) and reticulated aggregates (Fig. 10B). The stellate trillings are pseudohexagonal cyclical twins that are also commonly observed in many orthorhombic minerals, such as chrysoberyl and aragonite. Olivine trillings are scarcely reported in the literature; Burri (1935) documented several instances of twinned olivine in volcanic systems. The reticulated aggregate twin type is common in the orthorhombic mineral cerussite where crystals intersect in several directions to form a network. The pseudohexagonal symmetry exhibited by many orthorhombic minerals is due to the nearly hexagonal close-packing of anions; in the case of forsterite, it is the packing of oxygen atoms parallel to < 001 > .

Identifying the twin law responsible for the stellate trillings and reticulated aggregates is difficult because the twins could be reflections across either {110} or {310} or rotations around either < 110 > or < 310 > because the two sets of planes are orthogonal to one another. The dihedral angle between twin members is approximately 60 ± 0*.*5^*◦*^ for each law (Welsch et al. 2013; Azevedo and Nespolo 2017; Miller et al. 2021), which precludes discrimination via conventional EBSD procedure. The twin laws have exactly the same twin lattice but different twin planes. However, Azevedo and Nespolo (2017) found that the {310} twin ought to occur more frequently than the {110} based on the atomic topology of olivine. Hence, for the purpose of this manuscript, we refer to the two twins by their descriptive nomenclature (stellate trillings or reticulated aggregates) and do not attempt to assign a twin law to either type.

### Twinning frequency

It is well-known from industrial applications that the frequency of twinning generally increases with supersaturation, i.e., at higher supersaturations twins form more abundantly than they do at lower supersaturations (Klapper 2010, and references therein). However, this cannot be the only reason for the profusion of olivine twins because: [1] high olivine twin frequencies are not widely reported in highly supersaturated magmatic experimental and natural systems (cf., Donaldson 1976; Marquardt et al. 2015; Wieser et al. 2019) and [2] there is a positive correlation between bulk system Ca concentration and olivine twin frequency (Burri 1935; Larsen et al. 1941; Marquardt et al. 2015). The latter observation is intriguing, because it suggests that Ca substitution into olivine enhances the pseudohexagonal symmetry of the system by distorting the unit cell (e.g., Marquardt et al. 2015) *but this cannot be the case because the Ubehebe olivine has no appreciable Ca in it* (Fig. 5B and C).

One possibility is that Ca enrichment and lattice distortion only occurs at the twin boundary, as has been observed for Be in spinel with transmission electron microscopy (Daneu et al. 2007). In this system, Be is incorporated into the spinel twin plane during its initial formation and modifies the structure of the twin plane such that at the nanometer scale it is more similar to the polymorph taaffeite (Drev et al. 2013). The twin plane continues to grow and incorporate Be preferentially because the solubility of Be in the locally distorted lattice is higher than it is in the bulk of the crystal. Obviously, this does not result in a bulk Ca increase in olivine, and a focused TEM study is needed to confirm that this is also the case for twinned tabular forsterite.

### Unidirectional growth assisted by twin boundaries

The profuse twinning of olivine in our experiments and in the contact aureole reflects the large initial supersaturation conditions of forsterite nucleation and growth in both environments; the accident of twinning is more likely at high degrees of supersaturation (Klapper 2010, and references therein). It is also true that contact twinned crystals in general tend to: [1] be larger than co-existing single crystals [2] have elongate or flattened morphologies and [3] have faces that are usually absent from single crystals (Kitamura et al. 1979; Sunagawa et al. 1979). The twin growth systematics that give rise to these characteristics have been investigated in depth for quartz Japan Law $\{11\overline{2}2\}$ contact twins (Yasuda and Sunagawa 1982; Sunagawa and Yasuda 1983; Lee et al. 2002; Sunagawa et al. 2004), where it was concluded that flattened habits form as a consequence of the twin plane re-entrant edge effect (TPRE). The TPRE, sometimes called the apparent re-entrant edge effect, is the phenomenon of unidirectional growth along twin junctions due to dislocations in the twin plane. The twin boundary acts as a self-perpetuating step source so growth preferentially occurs in faces bounding the twin interface; in the case of twinned tabular forsterite, this growth occurs on the (100) face (Fig. 7). The importance of the TPRE as a general mechanism for the growth of faceted twinned crystals with otherwise unobserved morphologies has been confirmed experimentally in recent years for many different systems (Lee et al. 2002; Gamalski et al. 2014; Yu et al. 2014, 2010; Shahani et al. 2016).

An important aspect of TPRE is that it is less effective at higher supersaturation (Sunagawa et al. 1979). Hence, in order for a very large twinned crystals to form, they must nucleate or grow at a supersaturation that is sufficiently high to favor the formation of twinned nuclei (Buerger 1945; Hahn and Klapper 2006) but not at a supersaturation so high that growth occurs via an adhesive continuous growth mechanism; growth must occur below any roughening transitions. In other words, TPRE is only important for the precipitation of twinned faceted crystals growing by way of the 2D nucleation and growth or spiral growth mechanism. One implication of this is that in order for tabular forsterite to grow via the TPRE, the supersaturation must decrease rapidly after nucleation of twinned crystals.

### Kinetics of the Ubehebe Peak contact aureole

By incorporating the results of our experimental study with field observations, we can place some first order constraints on the kinetics of forsterite precipitation across the Ubehebe Peak contact aureole and explain the spatial distribution of olivine morphology and crystal size.

Roselle et al. (1997) concluded that the change in crystal size across the contact aureole was due to variation in nucleation rate relative to growth rate. Close to the intrusion where reaction overstepping is large, the nucleation rate is fast relative to the growth rate and so many small crystals form. Farther from the intrusion where reaction overstepping is small, the nucleation rate is slow relative to the growth rate and so a few large crystals form (Müller et al. 2009).

Our study supports the hypothesis from Roselle (1997) and Ferry (2011) that tabular olivine formation occurs in highly overstepped conditions and also identifies a growth mechanism that could be responsible for the morphology. The nucleation and initial growth of twinned tabular forsterite occurs by the highly overstepped consumption of quartz and dolomite throughout the contact aureole. At higher temperature in the inner contact aureole, subequant polyhedral overgrowths on tabular forsterite scaffolds form as tremolite reacts with dolomite. Tabular forsterite dissolves as further subequant polyhedral forsterite precipitation takes place. At lower temperature in the outer contact aureole, tabular forsterite grows into large crystals because supersaturation rapidly decreases immediately after nucleation and initial growth so that as growth proceeds at slower rates due to tremolite dissolution, the TPRE becomes the dominant growth mechanism. After a stable twinned crystal has formed, diffusion-limited growth is expected to further enhance the morphology of cm-scale crystals.

### Formation of subequant polyhedral forsterite

Within this kinetic framework, subequant polyhedral forsterite is found only close to the intrusion because that is the part of the contact aureole that was wet and that stayed the hottest for the longest. A high nucleation rate relative to growth would initially create many small tabular crystals which then recrystallize to subequant polyhedral forsterite via a textural equilibration process (Holness et al. 1989). Because the dissolution of tabular forsterite to precipitate subequant polyhedral forsterite is a relatively slow reaction, sustained high temperatures are necessary to allow the morphological conversion to run to completion. In addition, because magmatic-hydrothermal fluids fluxing out of the intrusion can have a strong component of vertical flow (Gerdes et al. 1995; Roselle et al. 1997; Nabelek 2002, 2007; Ferry et al. 2013) the inner contact aureole was probably more water-rich than the outer contact aureole. By tracking carbon and oxygen isotopic shifts, Roselle et al. (1997) showed that the direction of fluid flow in Ubehebe was mostly sub-vertical and that the high permeability infiltration pathways had a tube-like fingered geometry. The country rock had lost both water and porosity during the regional metamorphic event that had preceded the emplacement of the quartz monzonite, so when the magmatic fluids finally penetrated the pre-heated Lost Burro formation the forsterite-in reaction was highly overstepped.

The boron enrichment of the subequant polyhedral forsterite relative to the tabular also reflects some magmatic fluid input. We correlate the voids seen in experiments (Fig. 6C and 8C1) with the calcite and fluid inclusions observed in the Ubehebe samples.

### Driving force for dissolution of tabular forsterite

The dissolution of tabular forsterite and its recrystallization as subequant polyhedral grains, Reaction (5), is the slowest of the reactions occurring in the capsules and presumably in the contact aureole. There are two plausible reasons for the instability of tabular forsterite: (1) incorporated impurities have modified the solubility of tabular forsterite or (2) structural disorder. We exclude the first possibility that impurities in tabular forsterite are the driving force for Reaction 5 because Ubehebe subequant polyhedral forsterite is systematically enriched in impurities (e.g., boron) relative to Ubehebe tabular forsterite. Structural disorder in tabular forsterite is the likeliest driving force for Reaction 5. Structural disorder in tabular forsterite is evidenced by the more variable bond lengths and angle variances measured by XRD (Tables 1 and 2). This disorder is likely inherited from the initial extremely rapid growth kinetics, and is likely attended by a high concentration of crystallographic defects.

### Tremolite dissolution

Although there is not a well-developed tremolite zone in Ubehebe, it is possible that tremolite formed as a precursor phase in what is now the very well-developed forsterite zone. Similarly, to what is observed in our experiments, this tremolite would have been transient and dissolved almost immediately to form olivine.

If tremolite precipitated before or contemporaneously with forsterite in the contact aureole, then tremolite dissolution would contribute to slow tabular growth instead of the formation of a a subequant polyhedral overgrowth because the rate of tremolite dissolution reaction would be slower in the outer contact aureole than in the inner due to lower temperatures. Diffusion of elements from dissolving tremolite to precipitating forsterite further contributed to the diffusion-limited growth of very large tabular forsterite. Calling on Reaction (4) to precipitate large crystals of tabular forsterite in the outer contact aureole explains the petrographic observation that tabular forsterite is often in contact with unreacted dolomite because no dolomite is involved in Reaction (4). There is no evidence of extensive tabular forsterite dissolution in the outer contact aureole.

## Conclusions

In the Ubehebe Peak contact aureole, high nucleation rates close to the intrusion originally form many small crystals of tabular olivine. Farther out, where the contact aureole is cooler and less wet, the nucleation rate is slower and the growth rate is faster so tabular olivine crystals are bigger. Over time, the small tabular crystals in the hot, wet inner aureole recrystallize into small subequant polyhedral crystals. The tabular morphology is a consequence of twinning, which is widespread throughout the contact aureole due to high supersaturations. The spatial distribution of olivine shape and size is due to complex disequilibrium reaction pathways, which were observed in an experimental time series. Variation in mineral morphology, structure, and geochemistry in marbles is typically interpreted to reflect changes in system composition or temperature, but we have shown that reaction overstepping/supersaturation alone can influence the kinetics of nucleation and growth to create spatial distributions in crystal shape. We have identified a twin-mediated growth mechanism that could give rise to the unique habit of tabular forsterite, and shown that tabular forsterite is structurally disordered to some extent.

Fundamentally, disequilibrium gives rise to the remarkably rapid kinetics evinced by the results of our experimental time series and by the olivine textures recorded in Mg-marbles. Reaction overstepping causes the formation of twinned, structurally disordered tabular forsterite via the metastable consumption of quartz and dolomite. The tabular morphology of Ca-poor forsterite is an outcome of twinning at high supersaturation in a Ca-rich system. Similar processes likely occur in other natural environments, such as in undercooled magmas and regional metamorphism. Identifying criteria to fingerprint disequilibria in the natural record is necessary for the application of equilibrium thermodynamics to petrogenesis and will ultimately allow for disequilibrium textures to inform petrologic timescales.

## Data Availability

The following data are available for download in the Supplementary Material: EPMA and LAICPMS geochemical data, representative Raman spectra, and the crystallographic information files from XRD analysis.
